# Risk factors and outcomes of urosepsis in patients with calculous pyonephrosis receiving surgical intervention: a single-center retrospective study

**DOI:** 10.1186/s12871-019-0729-3

**Published:** 2019-05-01

**Authors:** Xia Liang, Jiangju Huang, Manyu Xing, Liqiong He, Xiaoyan Zhu, Yingqi Weng, Qulian Guo, Wangyuan Zou

**Affiliations:** Department of Anesthesiology, Xiangya Hospital, Central South University, 87 Xiangya Road, Changsha, 410008 Hunan China

**Keywords:** Calculous pyonephrosis, Sepsis, Upper urinary calculi, Urosepsis

## Abstract

**Background:**

Urosepsis is a catastrophic complication, which can easily develop into septic shock and lead to death if not diagnosed early and effectively treated in time. However, there is a lack of evidence on the risk factors and outcomes in calculous pyonephrosis patients. Therefore, this study was conducted to identify risk factors and outcomes of intra- and postoperative urosepsis in this particular population.

**Methods:**

Clinical data of 287 patients with calculous pyonephrosis were collected. In the univariate and multivariate analysis, all patients were divided into urosepsis group and non-urosepsis group. The diagnosis of urosepsis was mainly on the basis of the criteria of American College of Chest Physicians (ACCP)/Society of Critical Care Medicine (SCCM). Patient characteristics and outcomes data were analyzed, and risk factors were assessed by binary logistic regression analysis.

**Results:**

Of 287 patients, 41 (14.3%) acquired urosepsis. Univariate analysis showed that white blood cell (WBC > 10*10^9/L) before surgery (*P* = 0.027), surgery types (*P* = 0.009), hypotension during surgery (*P* < 0.001) and urgent surgery (*P* < 0.001) were associated with intra- and postoperative urosepsis for calculous pyonephrosis patients. In multivariate analysis, hypotension during surgery and urgent surgery were closely related to intra- and postoperative urosepsis. Outcome analysis suggested that patients developing urosepsis had a longer intensive care unit (ICU) stay and postoperative hospital stay and higher mortality.

**Conclusions:**

Hypotension during surgery and urgent surgery were risk factors of intra- and postoperative urosepsis for calculous pyonephrosis patients, which may lead to a prolonged ICU stay, postoperative hospital stay and higher mortality.

## Background

Sepsis and severe sepsis are life-threatening conditions encountered by clinicians, posing a huge burden on society [1–8]. Previous findings clarified that the incidence of sepsis has been increasing in recent 30 years [9], and the incidence of severe sepsis or septic shock ranged from 8.7 to 19.7%, with a high mortality rate ranging from 30.8 to 42% [2, 10–12]. Urosepsis refers to sepsis due to infection of the urinary tract and/or male reproductive system [13]. It has been reported that approximately 215,000 patients died of severe septic shock in the United States every year, and 9.1% of the infections originate in the genitourinary system, far below respiratory system (44.0%) and bacteria (17.3%) [4]. Thus, urosepsis is prone to be overlooked for a lower incidence and a better prognosis than pneumonia associated sepsis by inexperienced clinicians [4, 14] and then develop into septic shock and cause unexpected fatal events.

Calculous pyonephrosis is a common complication of urolithiasis and tends to progress into sepsis. It was reported that the prevalence of urolithiasis has increased in both Asian, European populations and the United States in recent decades, no matter in adult or pediatric patients [15–18]. Besides, many studies have demonstrated risk factors of developing urosepsis after percutaneous nephrolithotomy (PCNL) [19–23], however, there is no data on the risk factors and outcomes for the calculous pyonephrosis patients. Therefore, in the retrospective study, we aimed to investigate potential risk factors and outcomes of intra- and postoperative urosepsis in this particular population.

## Methods

### Study population

In this single-center retrospective study, we searched electronic medical records from our local Urology for urolithiasis patients having surgical treatment between January 1, 2015 and August 31, 2018 in Xiangya Hospital of Central South University. During the period, 4541 patients were searched. The criteria of exclusion included: (1) patients without upper urinary tract stones and without calculous pyonephrosis;(2) patients combined severe systemic disease that was a constant threat to life or even worse, that is American Society of Anesthesiologists (ASA) class IV-VI status;(3) patients underwent nephrectomy;(4) patients missing data. The definition of severe systemic disease that was a constant threat to life in our study mainly based on the ASA-Physical Status Classification System definitions and the newly ASA-approved examples [24]. Surgical patients with calculous pyonephrosis included were identified according to surgical record and X-ray findings, ultrasound or computed tomography (CT) data. Finally, 287 patients were enrolled and divided into urosepsis group and non-urosepsis group according to whether or not developing urosepsis.

### Data collection

All data were obtained from electronic records retrospectively except re-admission within 90 days. We mainly collected the following data: patient characteristics, preoperative co-morbidities, preoperative ASA classification, preoperative WBC and urine leukocyte (U-LEU), preoperative serum creatinine (Scr), stone size and types, surgery types and duration, intraoperative hemodynamics, postoperative ICU admission and ICU length of stay (LOS), total hospital LOS, postoperative hospital LOS and re-admission within 90 days. To reduce information bias, the data of re-admission within 90 days were gotten from electronic records as well as telephone follow-up. Urgent surgery in this study included urgent conditions which needed to remove obstruction, restore renal function and relieve refractory pain, such as patients complicated with high fever, low back pain, leukocytosis or decreasing of blood pressure and increasing of heart rate; acute upper urinary tract obstructive renal failure; acute renal colic (especially refractory renal colic) and so on.

### Diagnosis of urosepsis

Urosepsis refers to sepsis caused by infection of the urogenital tract [13]. In this retrospective study, we complied with the criteria of ACCP/SCCM [25, 26]. Patients enrolled were divided into urosepsis group if they were clinically diagnosed as urinary tract infection and had 2 or more signs of systemic inflammation response syndrome (SIRS), which include: temperature > 38.0 °C or < 36.0 °C; heart rate > 90/min; respiration rate > 20/min or PaCO_2_ < 32 mmHg or less (4.3 kPa); WBC count > 12,000/mm^3^, < 4000/mm^3^, or > 10% immature bands. Intraoperative urosepsis in this study was diagnosed through combining their intraoperative hemodynamic changes and proofs of preoperative, intraoperative and early postoperative infection (within 6 h after surgery). The criteria were as follows: (1) intraoperative hemodynamic changes, like hypotension, tachycardia and fever, which could not be corrected by symptomatic treatments, such as monitoring the depth of anesthesia, blood and/or fluids transfusion, or injection of dantrolene;(2) fluid resuscitation therapy and intravenous pumping vasoactive drugs were needed to maintain intraoperative and postoperative blood pressure; (3) proofs of perioperative infection, include leukocytosis, U-LEU positive or fever before surgery, pyonephrosis was found during surgery, procalcitonin > 0.5 ng/ml [27] or blood culture positive. Given incomplete data and our study purpose, we did not differentiate between sepsis, severe sepsis and septic shock, and not differentiate between intraoperative and postoperative sepsis for statistical analysis.

### Definition of intraoperative hypotension

The definition of intraoperative hypotension has remained controversial. Based on several previous studies [28–30], we have used these thresholds in our study: a systolic blood pressure (SBP) of 90 mmHg or less, or a reduction of 40 mmHg from pre-induction SBP baseline, or a mean arterial pressure (MAP) lower than 65 mmHg, or a decrease of 30% or more relative to the pre-induction MAP. When both invasive and noninvasive BP measurements were available, the invasive BP data were used.

### Statistical analysis

Categorical variables were reported using n (%); while continuous variables using means ± SD or median (P25, P75). Continuous variables were identified as a normal distribution by Kolmogorov-Smirnov test and Q-Q plot. Univariate analysis was performed using the chi-square test or Fisher’s exact test for categorical variables, and unpaired t-test or Mann-Whitney test for continuous variables. In the multivariate analysis, all variables in the univariate analysis were considered as independent variables and a binary logistic regression model was applied (entering and removing probabilities are 0.05 and 0.10 respectively). For all analyses, variables were considered statistically significant when *P* < 0.05 (two-sided). All statistical analyses were performed using SPSS 23.0 for Windows.

## Results

### Patient characteristics

**Fig. 1:**
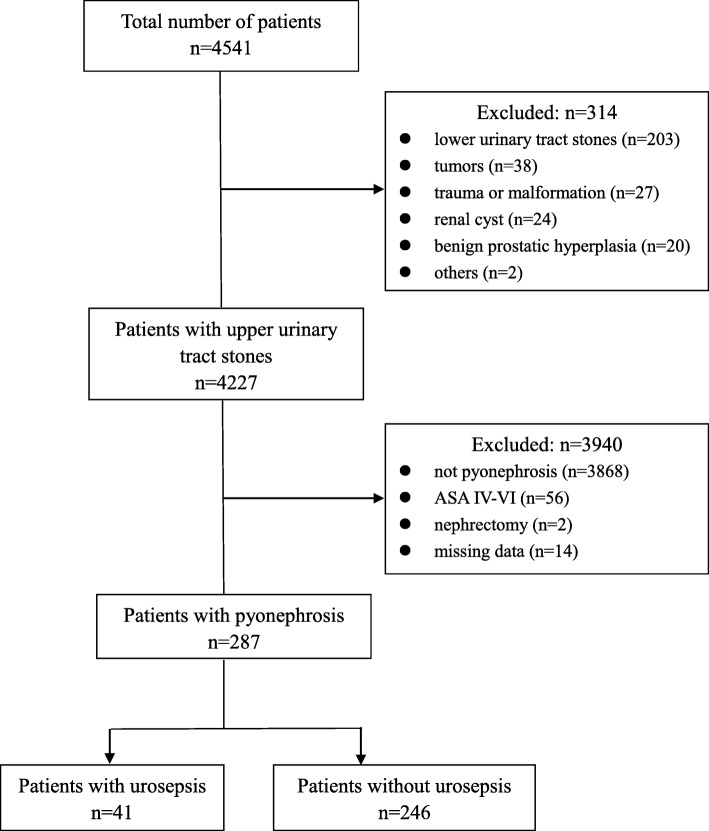
The flow diagram to illustrate the enrollment of study population

During the study period, a total of 4541 patients were admitted to our Department of Urology for surgical treatment. Of these, 4227 were diagnosed as upper urinary tract stones. We excluded 3940 patients according to exclusion criteria; thus, 287 patients with calculous pyonephrosis were included in the further analysis (Fig. 1). Based on the criteria of diagnosis mentioned, 41 (14.3%) patients were identified as urosepsis (Urosepsis group, 12 as intraoperative urosepsis and 29 postoperative urosepsis), and 246 (85.7%) patients were not (Non-urosepsis group).

Patient characteristics are shown in Table 1. The average age of the enrolled 287 patients (108 men and 179 women) was 53.81 ± 11.89 years. Apart from ASA classification (*P* = 0.002), there was no statistical significance between the two groups of other characteristics, which demonstrates that patient characteristics were almost equally distributed among patients with and without urosepsis.

**Table 1:** Patient characteristics of non-urosepsis and urosepsis groups

	Total (*n* = 287)	Urosepsis group (*n* = 41)	Non-urosepsis group (*n* = 246)	*P* value
Age (yr)	53.81 ± 11.89	53.80 ± 11.18	53.81 ± 12.02	0.997
Females	179 (62.4%)	28 (68.3%)	151 (61.4%)	0.398
Rural	223 (77.7%)	36 (87.8%)	187 (76.0%)	0.093
Education (yr)				
≤ 6	96 (33.4%)	16 (39.0%)	80 (32.5%)	0.704
16–12	160 (55.7%)	21 (51.2%)	139 (56.5%)	
≥ 12	31 (10.8%)	4 (9.8%)	27 (11.0%)	
Smoking	53 (19.6%)^a^	7 (17.1%)	46 (20.0%)	0.663
Comorbidity				
DM	28 (9.8%)	2 (4.9%)	26 (10.6%)	0.394
Hypertension	51 (17.8%)	4 (9.8%)	47 (19.1%)	0.147
CHD	12 (4.2%)	2 (4.9%)	10 (4.1%)	1.000
CKD	82 (28.6%)	12 (29.3%)	70 (28.5%)	0.915
ASA classification				
I-II	144 (50.5%)^a^	11 (27.5%)	133 (54.3%)	0.002**
III	141 (49.5%)^a^	29 (72.5%)	112 (45.7%)	

*DM* Diabetes mellitus, *CHD* Coronary heart disease, *CKD* Chronic kidney disease; ^a^Total number of patients is not 287 due to missing data; **P < 0.01

### Risk factors for intra- and postoperative urosepsis

We further determined what factors were related to an increased risk of acquiring urosepsis during and after surgery. In a univariate analysis, we analyzed potential predictors from three aspects of patient, stone and surgery. As is shown in Table 2, WBC before surgery, surgery types, hypotension during surgery and urgent surgery were associated with the occurrence of urosepsis (*P* < 0.05). In a multivariate analysis, only intraoperative hypotension and urgent surgery were independent predictors for patients with calculous pyonephrosis acquiring urosepsis (see Table 3). Surprisingly, factors related to the patients such as age, sex, combining with diabetes mellitus, U-LEU and Scr before surgery, as well as stone such as stone size and types, and operation time were neither in the univariate nor in the multivariate analysis significantly associated with urosepsis (*P* ≥ 0.05).

**Table 2:** Univariate analysis of variables associated with urosepsis during and after surgery

Variables	Urosepsis group (*n* = 41)	Non-urosepsis group (*n* = 246)	*χ2* value	*P* value
Age > 65 yr	6 (14.6%)	42 (17.1%)	0.611	0.435
Females	28 (68.3%)	151 (61.4%)	0.715	0.398
DM	2 (4.9%)	26 (10.6%)	0.727	0.394
WBC^a^ > 10 (10^9/L)	12 (36.4%)	46 (19.5%)	4.873	0.027*
U-LEU^a^ positive	27 (96.4%)	198 (90.0%)	0.576	0.448
Scr^a^ > 178 (umol/L)	13 (39.4%)	67 (28.6%)	1.596	0.206
Stone size > 2 (cm)	8 (21.6%)	75 (33.0%)	1.924	0.165
Multiple stones	35 (85.4%)	226 (91.9%)	1.101	0.294
Surgery types				
PCNL+URL^b^	28 (68.3%)	209 (85.0%)	6.785	0.009**
Others^c^	13 (31.7%)	37 (15.0%)		
Surgery duration > 120 (min)	4 (9.8%)	28 (11.4%)	0.001	0.969
Hypotension during surgery	13 (31.7%)	20 (8.2%)	16.837	< 0.001***
Urgent surgery	25 (61.0%)	28 (11.4%)	57.406	< 0.001***

*DM* Diabetes mellitus, *WBC* White blood cell, *U-LEU* Urine leukocyte, *Scr* Serum creatinine; ^a^data before surgery; ^b^Percutaneous nephrolithotomy (PCNL) and/or ureteroscopic lithotripsy (URL); ^c^Surgery types excluded PCNL and URL; *P < 0.05; **P < 0.01; ***P < 0.001

**Table 3:** Multivariate analysis of variables associated with urosepsis during and after surgery

	B	S.E.	*Wald* value	*P* value	OR	95% CI bounds	
						Lower	upper
Urgent surgery	2.220	0.515	18.553	< 0.001***	9.210	3.353	25.295
Hypotension during surgery	1.804	0.505	12.778	< 0.001***	6.076	2.259	16.342

*B* Regression coefficient, *S. E.* Standard error, *Wald* value Wald *χ*^*2*^ (test statistics); *OR* Odds ratio, *CI* Confidence intervals; ***P < 0.001

### Outcomes variables

As urosepsis is regarded as a critical perioperative complication in stone patients, we correlated its incidence with several outcome parameters. The occurrence of urosepsis was closely associated with a remarkably poor prognosis (see Table 4). Patients who developed urosepsis had a prolonged ICU length of stay and postoperative hospital LOS as well as higher mortality. In addition, there was no significant difference of total hospital LOS and re-admission within 90 days between urosepsis and non-urosepsis groups.

**Table 4:** Outcome characteristics of non-urosepsis and urosepsis groups

	Total (*n* = 287)	Urosepsis group (*n* = 41)	Non-urosepsis group (*n* = 246)	*P* value
ICU LOS >3 (days)	19 (6.6%)	15 (36.6%)	4 (1.6%)	< 0.001***
^a^Total hospital LOS (days)	9 (7,11)	8 (5.5,10)	9 (7,11)	0.076^b^
^a^Postoperative hospital LOS (days)	5 (4,7)	7 (4.5, 8.5)	5 (4,7)	0.022*^b^
Death	4 (1.4%)	4 (9.8%)	0 (0%)	< 0.001***
Re-admission within 90 days	122 (42.5%)	14 (34.1%)	108 (43.9%)	0.242

*LOS* Length of stay; ^a^variables described as median (P25, P75) for they did not form a normal distribution by Kolmogorov-Smirnov test and Q-Q plot; ^b^*P* value by Mann-Whitney test; *P < 0.05; ***P < 0.001

## Discussion

The main results of this retrospective study included: (1) urosepsis is a common complication for surgical patients with calculous pyonephrosis; (2) intraoperative hypotension and urgent surgery were risk factors for developing urosepsis during intra- and postoperative periods; (3) urosepsis during and after surgery prolonged ICU LOS and postoperative hospital LOS as well as increased mortality, which remarkably suggested a worse prognosis. Although there are quite a few studies to explore risk factors of urosepsis for patients with stones after PCNL or ureteroscopy (URS) [19–23, 31–33], to our knowledge, this is the first clinical retrospective study in the population of surgical patients with calculous pyonephrosis.

Besides, we did obtain some different results from previous research. Urosepsis is mainly caused by obstructive lesions of upper urinary tract, of which urolithiasis is the most common cause. A prior study reported that the incidence of urosepsis and septic shock after PCNL was 0.3–2.5% [21], however, little is known about patients with calculous pyonephrosis. While in the single institution study, we got an apparent higher rate of 14.3% of developing urosepsis in this particular population. The characteristic may be the reason that patients with calculous pyonephrosis are often more serious in the renal infection than most patients with urolithiasis undergoing PCNL.

One of the main objectives of this study was to investigate risk factors of urosepsis in calculous pyonephrosis patients with particular emphasis to variables describing patient, stone and surgery, however, contrary to what we expected, most of patient characteristics as well as stone features were not associated with urosepsis during and after surgery. This result was unexpected due to what some persuading evidence reported [13, 23, 34]. Wagenlehner and colleagues [13] believed that urosepsis tends to occur in elderly patients with a history of diabetes mellitus and immunosuppressed patients, such as those receiving organ transplants or chemotherapy or corticosteroids treatment, and those with acquired immunodeficiency syndrome. In addition, it was reported that women was an independent risk factor for urosepsis after PCNL [23, 34], and the incidence of bacteriuria, urinary tract infections and sepsis in women after PCNL was 2 times higher than that of men [35]. While in this study, we found that neither elderly patients nor women did increase the risk of postoperative urosepsis in patients with calculus pyonephrosis. With respect to the stone features increasing risk of urosepsis, previous literatures have shown that infectious stones, stones with a diameter more than 2.5 cm or a surface area greater than 10 cm^2^, stones in the renal pelvis or with obstructive lesion were potential predictors for postoperative urosepsis [22, 23, 36, 37]. However, in our study, multivariate logistic regression analysis showed that stone size and types were not independent risk factors for postoperative urosepsis. We believe those are because the study population was different from prior evidence. We mainly focused on calculous pyonephrosis patients while most of the previous ones concentrated on stone patients undergoing PCNL or URS. Furthermore, given that the sample size might have been too small to identify a correlation between those variables and risk for urosepsis occurrence, our results should be interpreted with caution. Hence, larger studies including more patients are needed for further investigation.

In addition, through multivariate analysis, we found that for patients with calculus pyonephrosis, intraoperative hypotension and urgent surgery may increase the risk of urosepsis, which were not surprising. Intraoperative hypotension was one of criteria we identified intraoperative urosepsis, which means that it is very likely to be associated with the occurrence of urosepsis. Taken into account insufficient preparation before surgery and uncertainty during urgent surgery, that urgent surgery increased risk for urosepsis seem to be reasonable. More importantly, in our study, we found many patients undergoing urgent surgery had obstructive lesions, which per se is the cause of urosepsis. Therefore, for those who do need urgent surgery, especially those who need to relieve obstruction, the severity and complexity should be fully realized and urologists should make the optimal judgment during surgery.

As for outcomes characteristics of urosepsis group and non-urosepsis group, as we expected, patients with calculous pyonephrosis who acquired urosepsis had a longer ICU LOS and postoperative hospital LOS and higher mortality than those did not acquire urosepsis. By further analysis, we found an interesting phenomenon that a large proportion of patients with urosepsis underwent urgent surgery, rather than elective surgery, resulting in shorter preoperative preparation time, which might be a possible explanation of the total hospital LOS with no significant difference but a longer postoperative hospital LOS.

Some limitations of our research have to be noted. As we mentioned, one major limitation is deriving from the small sample size, which may contribute to some false negative results inconsistent with previous data. The single-center retrospective design is another important weakness, making it difficult to interpret more internationally and avoid bias completely. Besides, the retrospective design and the new definition of intraoperative urosepsis may lead to an inaccurate estimation of urosepsis occurrence. Moreover, limited to the study design and missing data, we did not analyze more risk factors such as preoperative midstream urine culture and some biomarkers like C-reactive protein, procalcitonin and intraoperative perfusion pressure, which previous studies have shown a correlation with the risk of sepsis [19, 27, 38–42]. Combining these weak points, our analyses should be interpreted prudently and further multi-center randomized controlled studies including a larger case number are needed.

Despite these limitations, we still hope to emphasize the high incidence and adverse effect of urosepsis on calculous pyonephrosis patients and come up with some suggestions. One proposal is to fully prepare before urgent surgery. Another important step is that urologists should be better realize the severity and complexity of urosepsis and minimize operation time as possible, sometimes a staged procedure is recommended [18]. Furthermore, if necessary, it is advisable for anesthesiologist to focus more on vital signs of patient and remind the chief surgeon to transfer the patient to the ICU earlier according to the patient’s intraoperative conditions because 90% septic complications occur in the early postoperative phase [43]. Given that patient safety has not yet improved evidently for specialties including surgery, ICU and anesthesiology recently [44], multidisciplinary collaboration between anesthesiologists, urologists and ICU physicians might be beneficial for improving outcome.

## Conclusions

We found a high incidence of intra- and postoperative urosepsis in surgical patients with calculous pyonephrosis. Intraoperative hypotension and urgent surgery were independent variables associated with urosepsis during and after surgery, which may pose a heavier burden on patients due to a prolonged ICU LOS and postoperative hospital LOS. Larger prospective studies are needed to further investigate the risk factors and prognosis of urosepsis for this patient population.

## Abbreviations

ACCP: American College of Chest Physicians
ASA: American Society of Anesthesiologists
ICU: Intensive care unit
LOS: Length of stay
MAP: Mean arterial pressure
PCNL: Percutaneous nephrolithotomy
SBP: Systolic blood pressure
SCCM: Society of Critical Care Medicine
Scr: Serum creatinine
SIRS: Systemic inflammatory response syndrome
U-LEU: Urine leukocyte
URS: Ureteroscopy
WBC: White blood cell
